# Impact of Moderate Sedation versus Monitored Anesthesia Care on Outcomes and Cost of Endobronchial Ultrasound Transbronchial Needle Aspiration

**DOI:** 10.1155/2019/4347852

**Published:** 2019-05-09

**Authors:** Ziad Boujaoude, Rohan Arya, Aseem Shrivastava, Melvin Pratter, Wissam Abouzgheib

**Affiliations:** ^1^Division of Pulmonary and Critical Care Medicine, Cooper Medical School of Rowan University, Camden, NJ 08103, USA; ^2^Division of Pulmonary and Critical Care Medicine, University of South Carolina School of Medicine, Columbia, SC 29203, USA; ^3^WellStar Pulmonary Medicine, Marietta, GA, USA

## Abstract

**Background and Objectives:**

The ideal type of sedation for endobronchial ultrasound transbronchial needle aspiration (EBUS-TBNA) is not known. Two previous studies comparing the diagnostic yield between moderate sedation (MS) and deep sedation/general anesthesia (DS/GA) had provided conflicting results with one study clearly favoring the latter. No study had addressed cost. This is concerning for pulmonologists without routine access to anesthesia services. Our objective was to assess the impact of MS and Monitored Anesthesia Care (sedation administered and monitored by an anesthesiologist) on the outcomes and cost of EBUS-TBNA.

**Materials and Methods:**

We performed a retrospective review of prospectively collected data on consecutive EBUS-TBNA performed under two different types of sedation in a single academic center. A diagnostic TBNA was defined as an aspirate yielding any specific diagnosis or if subsequent surgery or follow-up of nondiagnostic/normal aspirates showed no pathology. Current Medicare time-based allowances were used for professional charges calculation.

**Results:**

There was no difference observed between MS and MAC in regards of the diagnostic yield (92.9% versus 91.9%), procedure duration, number, location, and size of lymph node (LN) sampled, but there were more passes per LN with MAC. The average charges were 74.30 USD for MS and 319.91 for MAC. There were more hypotensive and desaturations episodes with MAC but none required escalation of care.

**Conclusions:**

When performed under MS, EBUS-TBNA has similar diagnostic yield as under MAC but may be associated with less side effects. The difference in sedation cost is modest; however, an additional 245$ for each EBUS done under MAC would have significant cost implications on the health system. These findings are of critical importance for bronchoscopists without routine access to anesthesia services and for optimization of healthcare cost and resource utilization.

## 1. Introduction

Bronchoscopy is one of the most common procedures performed by chest physicians. It is generally an uncomfortable procedure and the use of analgesia and sedation is recommended to enhance patient satisfaction and achieve optimal procedural conditions for physicians. Sedation technique varies among practitioners, institutions, and locations with a continuum ranging from minimal sedation to general anesthesia (GA). The majority of bronchoscopies in the United States are performed under either moderate or deep sedation. Moderate sedation (MS) is defined as a drug-induced depression of consciouness during which patient maintains spontaneous ventilation, cardiovascular function, and responsiveness to verbal or light tactile stimulus and no interventions are required to maintain a patent airway. During MS, the responsible physician typically assumes the dual role of performing the procedure and supervising the sedation. In contrast to MS, higher level of depression of consciousness is achieved during deep sedation (DS) and the ability to independently maintain ventilatory function and patent airway may be impaired. DS should be always administered by an anesthesiologist (American Society of Anesthesiologists advisory regarding privilege to nonanesthesiologist to perform procedures under deep sedation published in October 2017) and it is referred to as Monitored Anesthesia Care or MAC. During MAC, an anesthesia clinician continuously monitors and supports the patient's vital functiolns, administers sedative drugs and analgesics as needed, diagnoses and treats clinical problems that occur, and converts to GA if required [1].

Since its introduction in 2004, endobronchial ultrasound-guided transbronchial needle aspiration (EBUS-TBNA) has become one of the most important diagnostic tools available to chest physicians for the diagnosis of mediastinal and hilar lymphadenopathy, parabronchial structures, and lung cancer staging [2, 3]. It is associated with an overall median sensitivity of 89% and it is recommended as a first step sampling technique by the American College of Chest Physicians 2013 evidence-based practice guidelines [4]. The EBUS bronchoscope has a larger diameter than the regular bronchoscope and the staging procedure typically requires more time for complete sampling of the mediastinum. The choice of sedation in EBUS procedures is an important question for bronchoscopists as they seek the best conditions to optimize their diagnostic yield, enhance patient's satisfaction, and minimize complications. Sedation also represents a significant element in the economics of EBUS-TBNA as it relates to cost of care, work flow, and healthcare resources utilization.

The ideal type of sedation for EBUS has not been determined yet. The data is limited to three studies that compared MS and GA during EBUS [5–7]. Two of the three studies reported on diagnostic yield but had conflicting results with one study clearly favoring GA over MS [5, 6]. None of the studies reported on cost. These findings had generated great concerns for bronchoscopists who do not have routine access to General Anesthesia and Operating Rooms. EBUS procedures are performed in our institution in the bronchoscopy suite under either MS or MAC, depending on the availability of the anesthesiologist. An artificial airway (AA) is not used with MAC. This study's purpose was to assess the effect of all these variables on the diagnostic yield and complications rate from the perspective of a single center. We also attempted to determine the differences in cost associated with the use of the different sedation techniques.

## 2. Methods

The study protocol was approved by the Institutional Review Board at Cooper University Hospital, Cooper Medical School of Rowan University, Camden, New Jersey.

We performed a retrospective chart review of inpatients and outpatients who underwent EBUS-TBNA for evaluation of mediastinal and hilar lymphadenopathy from January 2012 to December 2013. Chart review continued until 100 consecutive cases were identified and included in each group. Only Patients who had cytopathology data were included. We excluded patients if an additional procedure was performed (radial EBUS, electromagnetic navigation, transbronchial biopsies, or therapeutic intervention). At our institution, and during that period of time, anesthesia services were only available on specific days. Patients scheduled on days when anesthesia was available received MAC, while those who had their procedures done on other days received MS. MAC sedation was administered by a certified registered nurse anesthetist (CRNA) under the supervision of a board-certified anesthesiologist. A combination of propofol, ketamine, midazolam, and fentanyl was used with no AA. MS was induced by boluses of midazolam and fentanyl administered by a registered nurse and managed by the operating physician. During both types of sedation, continuous electrocardiographic, pulse oximetry, respiratory rate, and intermittent blood pressure monitoring was provided. All procedures were performed in the bronchoscopy suite by an interventional pulmonologist and assisted by an interventional pulmonary fellow. EBUS-TBNA was performed with a real-time ultrasound bronchoscope (BF-UC-180F; Olympus Ltd., Tokyo, Japan) using a dedicated 22-gauge needle (NA-201SX; Olympus Ltd. All cases had rapid on-site cytological evaluation (ROSE)). Data collected included demographics, procedure duration (measured from the initial bronchoscope introduction until last bronchoscope removal), number, location, and size of lymph nodes (LN) stations sampled, number of passes per LN, postprocedure cytopathologic diagnosis, and complications related to procedure. Complications noted were hypotension (decrease in systolic BP below 90 requiring intervention), desaturations below 90% requiring intervention beyond the increase of FIO2 and jaw thrust maneuver, and escalation of care. A diagnostic TBNA was defined as an aspirate showing any specific diagnosis or if subsequent surgery or at least six months' imaging follow-up of nondiagnostic/normal aspirates showed no pathology. The diagnostic yield was defined as the percentage of subjects in whom EBUS-TBNA provided any specific definitive diagnosis. The professional cost for the administration of the sedation was calculated based on the current time-based Medicare allowances. This is separate from the professional cost for bronchoscopy, which is similar regardless of the type of sedation [8]. For the initial 15 minutes of MS services, the allowances are 57.93$. Thereafter, the services are billed in 15 minutes' increment, and the allowances are 12.47$ for every 15 minutes. MAC charges have a base unit of 6 for the first 15 minutes and then time is charged in 15 minutes' increments. The Medicare allowances for 6 units are 225.14$ and 75.05$ for every 15 minutes' increment. If there is a CRNA, the service is charged twice, but Medicare splits the bills and pays half of each bill.

For statistical analysis, independent* t*-tests were used to compare between the study groups for normally distributed continuous variables and Mann-Whitney tests were used to compare rank data for variables that were not normally distributed. We used chi-square tests for analysis of categorical data.

## 3. Results

Ninety-Nine patients were included in the analysis in each group as the follow-up data were not complete for one patient in each group. Baseline age and gender were similar. In the univariate analysis, and as expected, the dose of fentanyl was significantly higher in MS (median 100 (IQR 100-125) than MAC (median 50, IQR 8-60) (p<0.001)). There was also a significant difference between the use of medazolam with MAC (mean 1.25 (SD 0.92)) versus MS (mean 4.84 (SD1.97)). These are consistent with prior reports of mean dose to achieve MS [9]. There was no difference in the number, location, the average size, and the distribution of the diagnostic categories of the LN sampled. However, there were a higher number of passes per LN in MAC group. The average procedure duration was 30 minutes in both groups. The procedure and LN characteristics are shown in Table 1.

**Figure 1:**
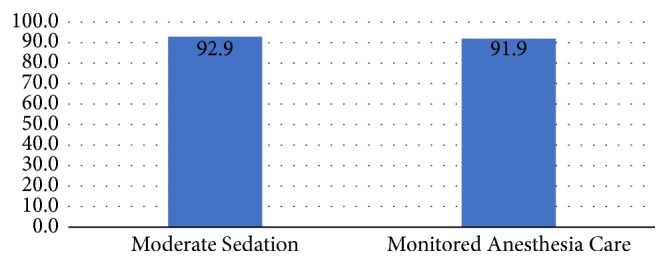
EBUS-TBNA diagnostic yield %.

**Figure 2:**
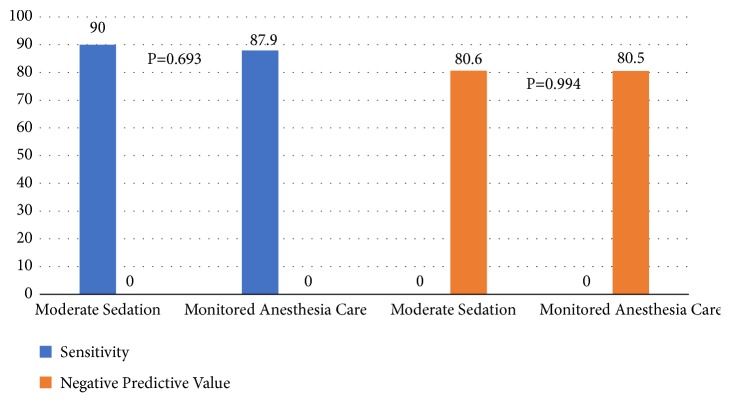
EBUS-TBNA sensitivity and negative predictive value.

There was no significant difference in the diagnostic yield between the two groups (92.9% MS versus 91.9% MAC, p = 0.788) (Figure 1). The diagnostic sensitivity and negative predictive values were comparable as well: sensitivity of 90% and 87.9% (p=0.693) and NPV of 80.6% and 80.5% (p=0.994) for MS and MAC, respectively (Figure 2).

**Figure 3:**
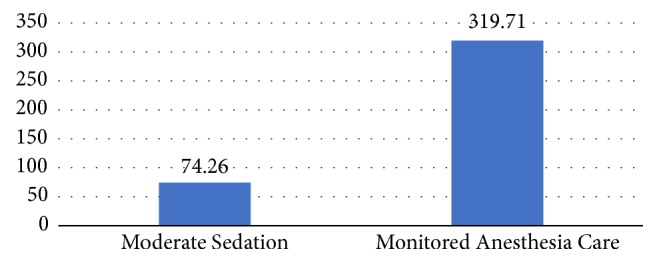
Average patient professional sedation charges in US dollars.

There were 15 patients who had relative hypotension in MAC group and none reported in the MS group. These hypotensive episodes were treated with boluses of phenylephrine as per the judgement of the anesthesiologist/CRNA. Outside of expected procedural desaturation easily corrected by minor interventions (9 patients requiring increasing FIO2 in MS group and 17 patients requiring Increasing FIO2 with or without jaw thrust in MAC group), none of the patients from either group developed significant hypoxemia that required escalation of care. In addition to baseline sedation charges and the first 15 minutes' increment, 31 patients had a second 15 minutes' increment charge in MS group and 26 in MAC group. The average sedation charges in USD were 74.26 and 319.71 per patient, respectively (Figure 3).

## 4. Discussion

The type of sedation remains an important question facing bronchoscopist performing EBUS-TBNA. Several factors impact the choice of sedation, including procedural outcomes, patient satisfaction, and cost. Unfortunately, the available data that attempted to answer this question is very limited, has shown conflicting results, and does not address differences in cost. This has led to a grade 2C recommendation by the ACCP 2016 guidelines on sedation aspects of EBUS-TBNA. The recommendation suggests that either moderate or deep sedation is an acceptable approach [10]. Furthermore, DS is performed with an AA and this requires the use of operating room (OR) in most instances as the majority of the bronchoscopy suites are not equipped for GA. This by itself generates concern among many pulmonologists in nontertiary settings where access to GA is limited or not available, and this without mentioning the significant implications on cost, work flow, and healthcare utilization.

To our knowledge, this is the first study that provides a direct comparison between EBUS-TBNA performed under MS or MAC in the bronchoscopy suite, without AA. This is also the first study that attempts to report on cost. We found that EBUS-TBNA is equally effective and safe when performed under either MS or MAC, with MAC associated with higher sedation professional cost.

Methods of sedation for EBUS-TBNA have been studied since its introduction in 2004, but most studies focused on safety and sedation tolerance [11–13]. Steinfort et al. concluded that EBUS-TBNA may safely be performed under conscious sedation and is associated with very high patient satisfaction. Ando et al. reported that EBUS-TBNA under the intravenous sedation by meperidine was as feasible and safe as that under GA. Sarkiss and coworkers described the performance of EBUS-TBNA under the use of propofol. They had no major complications but there was no data regarding diagnostic yield.

Similarly, the impact of various factors on EBUS-TBNA success rate and diagnostic yield has been extensively studied; however, only three studies reported on the impact of type of sedation on the diagnostic yield [5, 6, 14]. In Kennedy's study, factors such as lymph node size and location influenced the result but the procedure was performed under one type of sedation and the effect on the yield was not reported [14]. The two studies that provided a direct comparison of the effect of sedation on the yield between two groups were a retrospective study by Yarmus et al. and a randomized trial by Casal et al. [5, 6]. Both studies compared the diagnostic yield under MS and GA. These two studies reported conflicting results. Yarmus et al. reported a greater diagnostic yield when EBUS was performed under GA. This study was performed in two different centers, one performing all cases under MS and the other performing all cases under GA. This was the main limitation that was acknowledged by the authors. Performing the procedure in two different institutions can potentially lead to multiple confounding factors (different population, operators, and pathologist) that can influence the results. They also speculate that GA allows sampling of more LN and more needle passes per site leading to higher yield. In contrast to these findings, Casal and coworkers found no significant differences in diagnostic yield between the two groups. Also they found no difference in the number, size, and number of passes per LN. Our findings were more aligned with the findings of Casal's study in regards of the diagnostic yield and the number of LN stations sampled; however, there were more passes per LN in our MAC group.

Interestingly, the number of LN stations involved per patient has recently gained more attention with the publication of the Eighth Edition of Lung Cancer Stage Classification [15]. Subgroup analyses revealed separation in survival based on the number of unique LN involved in N1 and N2 stations [16]. The eighth edition makes a recommendation to quantify nodal disease by the number of involved nodal stations. This potentially will lead some bronchoscopist to favor deep sedation if they believe it will allow for more lymph node station sampling; however, the recommendation was clear in regards of the purpose, which is not to determine treatment options but rather should be considered together with patient-specific factors in clinical decision-making.

Another interesting finding in our study that could have potential effect on bronchoscopist decision is the higher number of passes per LN with MAC, a similar finding reported in the Yarmus study with GA. The number of passes per sampling site has potential implications on procedural efficiency, diagnostic yield, and sample adequacy for molecular testing. The ACCP 2016 guidelines suggest that a minimum of 3 separate needle passes be performed per sampling site to maximize diagnostic yield; however, this weak recommendation was based on only one study by Lee et al. [4]. In his study sample, adequacy was 90.1% after the first pass and 98.1% after two passes and reached 100% after three passes [17].

EBUS has been shown to have a very high adequacy rate for obtaining enough tissue to test for mutational markers [18], but only one retrospective study addressed the number of passes required [19]. A median of four passes, in conjunction with ROSE, was needed to establish an adequacy rate of 93.5% in this study. This data, as stated by ACCP 2016 guidelines, is insufficient to identify the number of passes needed to obtain adequate tissue for molecular marker testing. The higher number of passes with MAC should not be considered an argument for the routine use of MAC for EBUS.

None of the two studies by Yarmus and Casal reported on cost. This is an important factor to take into consideration when making choices for sedation technique. Our analysis shows that professional sedation charges are approximately 4 times higher with MAC. While the cost of MAC compared to MS may not be much different for a given case, a 245$ additional cost for each EBUS done under MAC would have significant cost implications for the US health system. A major advantage for the cost of MAC in our study is the absence of use of an artificial airway (whether a laryngeal mask airway or endotracheal tube). The artificial airway requires the use of the OR in most instances as the majority of the bronchoscopy suites are not equipped for GA, and this might increase significantly the cost and affect the availability and the work flow. The cost of EBUS in OR is comparable to mediastinoscopy [20].

Finally, we found no significant difference in the major complications rate associated with the two types of sedation; however, MS may potentially have less side effects (hypotension and desaturations).

This is in agreement with the majority of the published studies but is not aligned with the Quality Improvement Registry, Evaluation, and Education (AQuIRE) Data Registry finding of an association between GA and greater need for postprocedure escalation of care [21].

There are limitations to this study mainly related to the retrospective design; however, the study compared two similar groups during the same time period and was performed with same operators. Some bias concerning patient allocation is inevitable, although the decision to choose MAC versus MS was most often made by chance, mainly depending on whether the patient was scheduled on a day when anesthesia is available or not. This fact may have led to a pseudorandomization to this study.

Patient satisfaction was not addressed in the study; however, equal satsisfaction with both types of sedation has been repetitively shown in multiple studies as discussed earlier. Last, we could not report the rate of patients that could not tolerate or complete the procedure due to exclusion criteria.

In summary, EBUS-TBNA may be performed under moderate sedation without compromising the diagnostic yield and may potentially have less side effects. It is associated with less impact on the cost for the health system. This is of critical importance in this era where reducing expenses and optimizing resource utilization are becoming more and more essential.

MAC may have a potential to be superior in lung cancer staging and when more tissues are needed for molecular testing (sampling more LN stations and more passes per LN); however, the data is not sufficient to clearly favor this approach for these purposes and future research examining this effect will be valuable.

## Figures and Tables

**Table 1:** Patients and procedure characteristics.

	MS	MAC	P Value
**Number of patients**	**99**	**99**	

**Age (Mean/SD)**	62.45 (14.43)	60.38 (18.81)	0.387

**Sex - Male (n/**%**)**	46 (46.5%)	44 (44.4%)	0.775

**EBUS-TBNA diagnosis (n/**%**)**			0.536
*Adenocarcinoma*	29 (29.3%)	24 (24.2%)	
*Squamous Cell Carcinoma*	9 (9.1%)	15 (15.2%)	
*Sarcoid*	11(11.1%)	8 (8.1%)	
*Lymphoma*	5 (5.1%)	8 (8.1%)	
*Small Cell Carcinoma*	10(10.1%)	5 (5.1%)	
*Other*^*∗*^	6 (6.1%)	6 (6.1%)	
*Nondiagnostic/normal *	29 (29.3%)	33 (33.3%)	

**Procedure Time in minutes (Median/IQR)**	30 (23 – 40)	30 (22 – 38)	0.630

**Sedative dose**			

*Fentanyl (Median/IQR) *	100 (100 – 125)	50 (8 - 60)	< 0.001

*Midazolam (Mean/SD)*	4.84 (1.97)	1.25 (0.92)	<0.001

**Lymph node station sampled**			

** Total Lymph nodes (n/**%**)**	**226**	**204**	

*2R *	2 (0.9%)	3 (1.5%)	
*2L*	0 (0%)	1 (0.5%)	
*3P*	0 (0%)	1 (0.5%)	
*4R*	57 (25.2%)	42 (20.6%)	
*4L*	16 (7.1%)	11 (5.4%)	
*7*	74 (32.7%)	66 (32.4%)	
*8*	0 (0%)	2 (1%)	
*10R*	10 (4.4%)	9 (4.4%)	
*10L*	1 (0.4%)	2 (1%)	
*11R*	41 (18.1%)	31 (15.2%)	
*11L*	20 (8.8%)	32 (15.7%)	
*12R*	5 (2.2%)	4 (2%)	

Number of LN sampled/patient (Mean/SD)	2.29 (0.87)	2.06 (0.77)	0.048

Size of LN (Mean/SD)	18.88 (7.41)	18.03 (7.82)	0.440

Number of passes per LN	1.6 (0.6)	2.24 (0.91)	<0.001

^*∗*^The diagnoses that were included in “Other” were 6 breast cancers, 2 esophageal cancers, and one of each of the following: CLL, colon cancer, large cell carcinoma, or mesothelioma.

MS: moderate sedation; MAC: monitored anesthesia care; LN: lymph node.

## Data Availability

The data is securely stored in the database of the Pulmonary Division at Cooper University Hospital and is available upon request.
